# The Amount of Releasable Insulin Depends on Continuous Oxidative Phosphorylation

**DOI:** 10.1093/function/zqaf033

**Published:** 2025-07-23

**Authors:** Carolin Tappe, Manjitha Parambath, Julia Reschke, Ingo Rustenbeck

**Affiliations:** Institute of Pharmacology, Toxicology and Clinical Pharmacy, Technische Universität Braunschweig, D-38106 Braunschweig, Germany; Institute of Pharmacology, Toxicology and Clinical Pharmacy, Technische Universität Braunschweig, D-38106 Braunschweig, Germany; Institute of Pharmacology, Toxicology and Clinical Pharmacy, Technische Universität Braunschweig, D-38106 Braunschweig, Germany; Institute of Pharmacology, Toxicology and Clinical Pharmacy, Technische Universität Braunschweig, D-38106 Braunschweig, Germany

**Keywords:** cytosolic calcium concentration, insulin secretion, K_ATP_ channel, oxidative phosphorylation, pancreatic islets, plasma membrane potential

## Abstract

The consensus or canonical model of glucose-stimulated insulin secretion provides that the metabolism of glucose closes K_ATP_ channels by increase of the ATP/ADP ratio and that the ensuing depolarization-induced Ca^2+^ influx through voltage-dependent Ca^2+^ channels represents the immediate signal for the onset of exocytosis. However, it has been shown earlier that the depolarization-induced secretion can be suppressed by inhibition of the oxidative phosphorylation, pointing to an energy-requiring step presumably located downstream of Ca^2+^ influx. Here, we have investigated the relation between oxidative phosphorylation and the insulinotropic effect of K^+^ depolarization to better localize the energy-requiring step. The specific inhibitor of the mitochondrial F_1_F_O_ ATPase, oligomycin, concentration-dependently and time-dependently inhibited the insulin secretion elicited by a strong K^+^ depolarization (40 mm). Perifusion with 4 µg/mL of oligomycin for 20, 10, or 5 min prior to the K^+^ depolarization reduced the amount of insulin secreted from freshly isolated islets from control value to about 5% with a half-time of 1.6 min. 0.4 µg/mL of oligomycin required more time for comparable effects. Cultured islets were less susceptible to the inhibitory action of oligomycin than fresh islets, corresponding to their significantly higher ATP/ADP ratio. The perifusion with oligomycin prior to the K^+^ depolarization did not decrease the depolarization-elevated cytosolic Ca^2+^ concentration and did not affect the resting plasma membrane potential and the extent of depolarization by 40 mm KCl. In conclusion, the exocytotic machinery of the beta cell requires a continuously running oxidative phosphorylation to remain responsive to the Ca^2+^ signal for granule fusion.

## Graphical Abstract

**Graphical Abstract:**
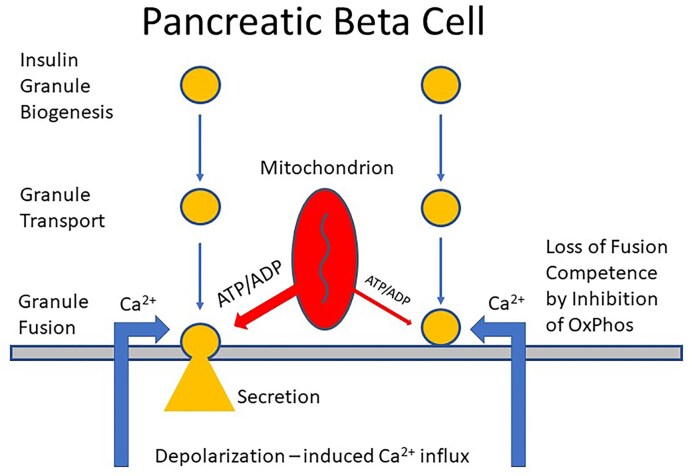

## Introduction

The consensus or canonical model of glucose-stimulated insulin secretion describes a linear sequence of events. First, the mitochondrial metabolism of glucose acts as the signal generator, since some amino- and keto-acids, which are directly fed into the Krebs cycle, can also stimulate insulin secretion.^1,2^ Second, the increase of the cytosolic ATP/ADP ratio as the result of activated oxidative phosphorylation closes ATP-sensitive K^+^ channels (K_ATP_ channels) and thereby enables the depolarization of the plasma membrane.^3,4^ Third, the influx of Ca^2+^ from the extracellular space through voltage-dependent Ca^2+^ channels (VDCC) delivers Ca^2+^ to the sites of insulin granule fusion.^5–7^ A vast number of experimental observations lend support to this model, for example, the inhibition of insulin secretion by inhibitors of oxidative phosphorylation,^8^ by K_ATP_ channel openers^9^ and by Ca^2+^ channel blockers.^10,11^

So, consistent with the SNARE model of triggered exocytosis,^12^ Ca^2+^ influx via VDCC is considered as the final common pathway for all those stimuli that do not only enhance but elicit insulin secretion. It has been suggested that the increase of Ca^2+^ in the submembrane space is restricted to act on granules that are docked at the plasma membrane and are functionally competent (primed) for release.^13,14^ Based on electrophysiological and ultrastructural data, these granules were termed the readily releasable pool.^13,15^ This pool of granules (which was subdivided later on) was proposed to form the first phase of glucose-stimulated insulin secretion.^16,17^ The observation that non-nutrient stimulation, such as K_ATP_ channel closure or depolarization by high extracellular K^+^ concentration, elicits a first phase-like secretion was regarded as evidence that the first phase does not depend on mitochondrial activity.^17,18^

Given the linear sequence of stimulus secretion coupling, it was unexpected that the inhibition of oxidative phosphorylation did not only abolish the insulinotropic effect of glucose but also of the depolarization by K_ATP_ channel block or high extracellular KCl.^19,20^ The depolarization-induced Ca^2+^ influx should have been sufficient to elicit secretion by the fusion of granules contained in the readily releasable pool. Therefore, it was presumed that the energy-requiring step was located distal to Ca^2+^ influx.^19,20^

In addition to the generation of electrical activity, the mitochondrial metabolism is the source of an additional signaling pathway, the perceived role of which is to amplify the secretory response to Ca^2+^ influx, but not to control the efficiency of the Ca^2+^ signal.^21^ While a number of signal mediators have been suggested to transmit the signals of metabolic amplification,^22^ the only generally accepted signal transducer between glucose metabolism and secretion is still the ATP/ADP ratio. Therefore, even sophisticated models of beta cell function (see eg,^23^) only consider the mitochondrial ATP production or the resulting changes of the cytosolic ATP/ADP ratio as regulator of the electrical activity and the ensuing insulin secretion.

Meanwhile, a number of observations confirm that the connection of depolarization-induced Ca^2+^ influx with insulin secretion comprises an energy-requiring step and that the K_ATP_ channel has not an exclusive role in connecting the oxidative phosphorylation with insulin secretion.^24,25^ Here, we investigated whether the energy-requiring step is really located distal to Ca^2+^ influx as originally proposed.^19,20^ To this end, the selective inhibition of oxidative phosphorylation by oligomycin was used to measure its effects on the secretion elicited by a strong depolarizing stimulus, the increase of the K^+^ concentration to 40 mm in the extracellular space. The resulting depolarization of the plasma membrane produces a fast and strong influx of Ca^2+^ and exerts a secretion stimulus of supraphysiological strength.^26,27^ The present observations suggest that the acute inhibition of oxidative phosphorylation lets the cytosolic level of ATP or the ATP/ADP ratio fall below a level that must be maintained to keep the exocytotic machinery responsive to the Ca^2+^ signal for granule fusion.

## Materials and Methods

### Chemicals

D600, oligomycin from Streptomyces diastatochromogenes (a mixture of oligomycin A, B, and C), nystatin, collagenase P, and the cell culture medium RPMI 1640 (without glucose) were from Sigma-Aldrich (Taufkirchen, Germany). Fetal calf serum (FCS Gold ADD) was obtained from Bio & Sell (Nürnberg-Feucht, Germany), bovine serum albumin (BSA, fraction V) and all other reagents of analytical grade were from E. Merck (Darmstadt, Germany).

### Ethical Approval

According to federal German law, the sacrifice of animals that have not been specifically treated nor are genetically altered does not constitute animal experimentation. Thus, only reporting but no formal permission is required. The central animal care facility of the TU Braunschweig (ZET) reports to the animal welfare authority of the state of Lower Saxony (LAVES, Oldenburg, Germany), and all its procedures conform to the current EU regulations.

### Islet Isolation and Tissue Culture

Islets were isolated from the pancreas of female NMRI mice (12–14 weeks old, fed ad libitum) by injection of 3 mL of a collagenase solution (0.75 U/mL) into the bile duct and incubation of the excised pancreas in a water bath for 9.5 min. After shaking by hand for 1 min, the islets released from the exocrine tissue were hand-picked under a stereomicroscope. The time between onset of the digestion and the start of the perifusion of freshly isolated islets was about 45 min. For a direct comparison between freshly isolated islets and cultured islets, the same batch of islets was split in 2. Islets were cultured in RPMI 1640 with 5 mm glucose and 10% FCS in a humidified atmosphere of 95% air and 5% CO_2_ at 37°C. The culture duration was 22 ± 1 h.

### Measurement of Insulin Secretion and Insulin Content

50 freshly isolated or cultured islets were pipetted into a purpose-made perifusion chamber (36°C) and perifused at 1 mL/min with a HEPES-buffered Krebs-Ringer medium (KR-medium), which was saturated with 95% O_2_ and 5% CO_2_ and contained (mm): NaCl 118.5, KCl 4.7, CaCl_2_ 2.5, KH_2_PO_4_ 1.2, MgSO_4_ 1.2, NaHCO_3_ 20, HEPES 10, BSA 0.2% w/v. The insulin content of the fractionated efflux was determined by ELISA according to the manufacturer’s protocol (Mercodia, Uppsala, Sweden).

### Islet Content of Adenine Nucleotides

15 freshly isolated or cultured islets were statically incubated to mimic typical perifusion conditions. After precipitation of the proteins and extraction of the adenine nucleotides, ATP was determined by use of the luciferase method as previously described.^28^ The ADP content of the extract was converted into ATP by the pyruvate kinase reaction, the difference between both measurements yielding the net ADP content. Because of the interindividual variations in the adenine nucleotide contents, the incubations were strictly performed in parallel, and comparison between cultured and fresh islets was only made with islets from the same isolation batch.

### Measurement of the Cytosolic Ca^2+^ Concentration ([Ca^2+^]_i_)

Cultured islets were incubated in Krebs-Ringer medium (5 mm glucose) with Fura-2 LeakRes (AM) at a concentration of 2 µm for 40 min at 37°C. Five islets were then inserted in a temperature-controlled perifusion chamber (35°C) on the stage of a Zeiss Axiovert 135 microscope equipped with a Zeiss Fluar (10x, 0.5 N.A.) objective. The islets were perifused with a HEPES-buffered Krebs-Ringer medium, which was saturated with 95% O_2_ and 5% CO_2._ The fluorescence of each islet (excitation at 340 or 380 nm, dichroic separation at 410 nm, emission 510 ± 40 nm) was recorded with a cooled CCD camera (Pursuit, Diagnostics Instruments, Sterling Heights, MI, USA) and evaluated using Visiview software (Visitron, Munich, Germany).

### Electrophysiological Recordings

The membrane potential and whole-cell currents of single beta cells were measured using the perforated-patch configuration.^29,30^ Pipettes were pulled from borosilicate glass (2 mm o.d., 1.4 mm i.d., Hilgenberg, Germany) by a 2-stage vertical puller (List Electronic, Darmstadt, Germany) and had resistances between 3 and 6 MΩ when filled with solution. The compositions of the bath and pipette media were as described earlier.^31^ Currents and voltages were recorded by an EPC 7 patch-clamp amplifier (HEKA, Lambrecht, Germany) under control of pClamp 6.03 software (Axon Instruments, Foster City, CA, USA), and low pass-filtered by a 4-pole Bessel filter at 2 kHz. Exposure was done by a slow bath perfusion system, mimicking the conditions of the secretion measurements (lag time between change of the bath solution and the new steady state in the bath ca. 1 min). Experiments were performed at room temperature (20°C–22°C). Data were analysed off-line using GraphPad Prism 5 software (GraphPad, La Jolla, CA, USA).

### Statistics

GraphPad Prism 5 software (GraphPad, La Jolla, CA, USA) was used for statistic calculations and non-linear curve fitting. If not stated otherwise, “significant” refers to *P* < .05, the specific statistical analyses are given in the figure legends.

## Results

### Dependence of the Depolarization-Induced Insulin Secretion on the Presence of Glucose

**Figure 1.**
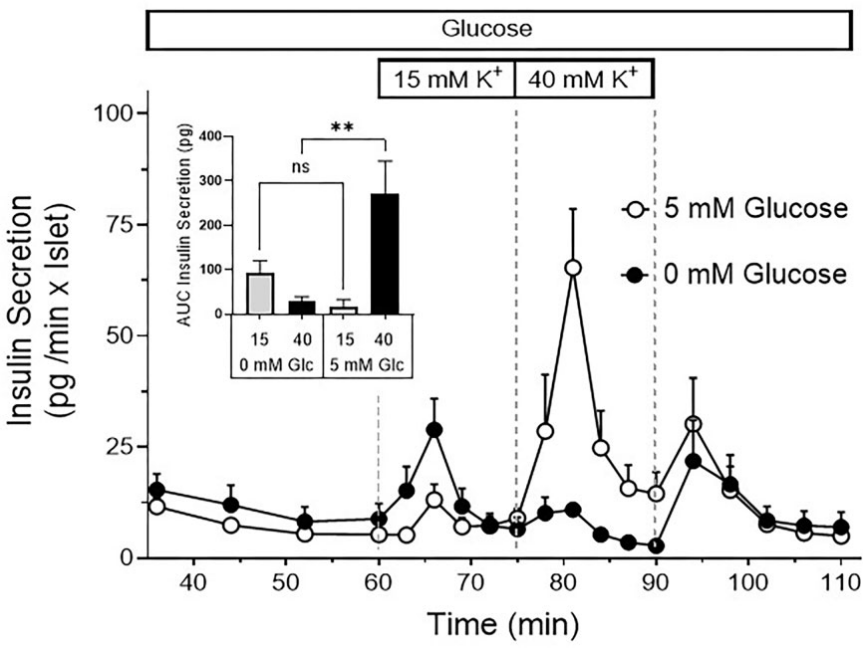
Nutrient dependency of the depolarization- induced insulin secretion. Freshly isolated islets were perifused with KR-medium (5 mm glucose) for 60 min, then the K^+^ concentration was raised from 5.9 to 15 mm for 15 min and increased further to 40 mm for another 15 min (open circles). Thereafter, the K^+^ concentration was lowered back to 5.9 mm. The same changes of the K^+^ concentration were made in the absence of glucose (closed circles). Note the virtual loss of effect of the second depolarization phase under this condition. The inset shows the integral of insulin released by K^+^ depolarization above the baseline rate. The comparison between 0 and 5 mm glucose (Glc) was calculated by one-way ANOVA with Sidak’s correction, 2 asterisks stand for *P* < .01, n.s. for non-significant. The values are means ± SEM of 5 experiments.

Increase of the K^+^ concentration from 5.9 to 15 mm in the perifusion medium of freshly isolated islets gave a modest monophasic increase of insulin secretion. The subsequent further increase to 40 mm resulted in an about 10-fold larger secretion (Figure 1). This response pattern was fundamentally altered when the glucose concentration of the perifusion medium prior to the depolarization was 0 mm instead of 5 mm. There were no significant differences in insulin secretion between 0 and 5 mm glucose in response to 15 mm KCl. Raising the K^+^ concentration further to 40 mm produced a strong increase in insulin secretion in the presence of 5 mm glucose, but only a minimal transient increase in the presence of 0 mm glucose (Figure 1). Irrespective of the glucose concentration, the washout of the high K^+^ concentration caused a transient increase of secretion before the respective prestimulatory secretion rates were re-established.

### Inhibitory Effect of Oligomycin on the Depolarization-Induced Insulin Secretion

**Figure 2.**
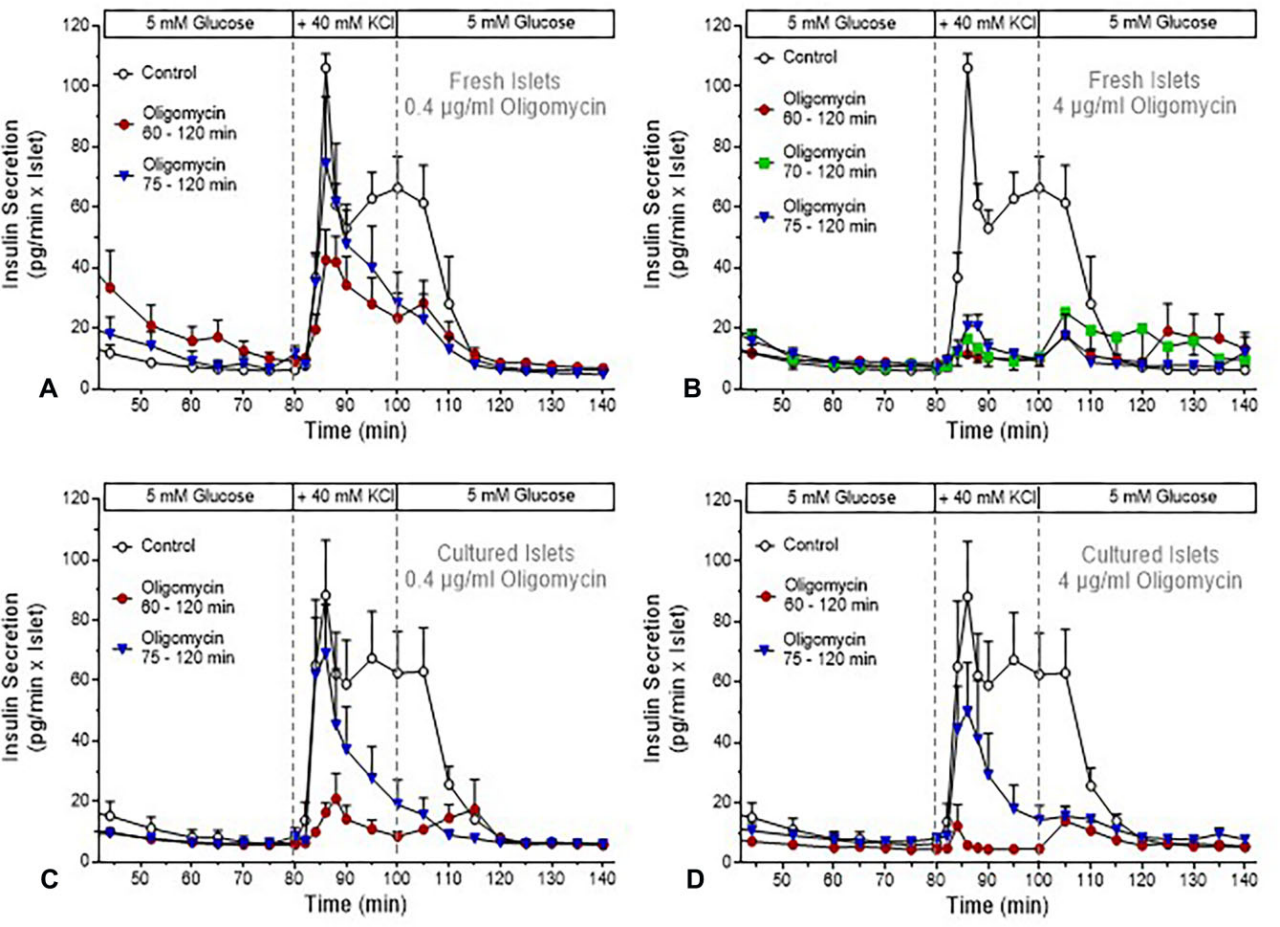
Inhibition of depolarization-induced insulin secretion by oligomycin. Freshly isolated islets were perifused with KR-medium (5 mm glucose) for 80 min, then the K^+^ concentration was raised from 5.9 to 40 mm for 20 min; thereafter, the K^+^ concentration was lowered back to the prestimulatory value (same control data in Figure 2A and B). This protocol was also used to stimulate 22 h-cultured islets (same control data in Figure 2C and D). The effect of oligomycin on the depolarization-induced insulin secretion was tested at 0.4 µg/mL (Figure 2A and C) or 4 µg/mL (Figure 2B and D). Oligomycin was present in the perifusion medium from 60 to 120 min (20 min pre-exposure, red circle symbols), 70 to 120 min (10 min pre-exposure, green square symbols, in Figure 2B only), or 75 to 120 min (5 min pre-exposure, blue triangle symbols). The values are means ± SEM of 5–8 experiments.

The inhibitory effect of oligomycin on the depolarization-induced insulin secretion by perifused fresh or cultured islets was tested at a high concentration (4 µg/mL) or at 10% thereof (0.4 µg/mL). The exposure ranged between 20 and 5 min prior to the increase of the K^+^ concentration and was maintained until 20 min after washout (Figure 2). While oligomycin at 0.4 µg/mL (Figure 2A and C) was less effective than at 4 µg/mL (Figure 2B and D) to inhibit the immediate response to K^+^ depolarization, it achieved a high degree of inhibition towards the end of the stimulation period.

When Oligomycin at 4 µg/mL was present for 20 min prior to the K^+^ depolarization of fresh islets, the stimulation of insulin was suppressed from the beginning on. Likewise, a 10 min exposure and even a 5 min exposure led to a strong inhibition (Figure 2B). At 0.4 µg/mL of oligomycin, the degree of inhibition was more dependent on the pre-exposure: the initial secretory response was halved after 20 min pre-exposure, but only marginally reduced by 5 min pre-exposure (Figure 2A).

When the effect of oligomycin on cultured islets was tested, the pre-exposure with 4 µg/mL for 20 min prior to the K^+^ depolarization suppressed the insulin secretion, but the 5 min exposure led only to a moderate reduction, a weaker effect than seen with fresh islets (Figure 2B vs 2D). At 0.4 µg/mL, the inhibitory effects of oligomycin on cultured islets corresponded to those on fresh islets in that a 20 min exposure was needed to diminish the initial response to K^+^ depolarization.

**Figure 3.**
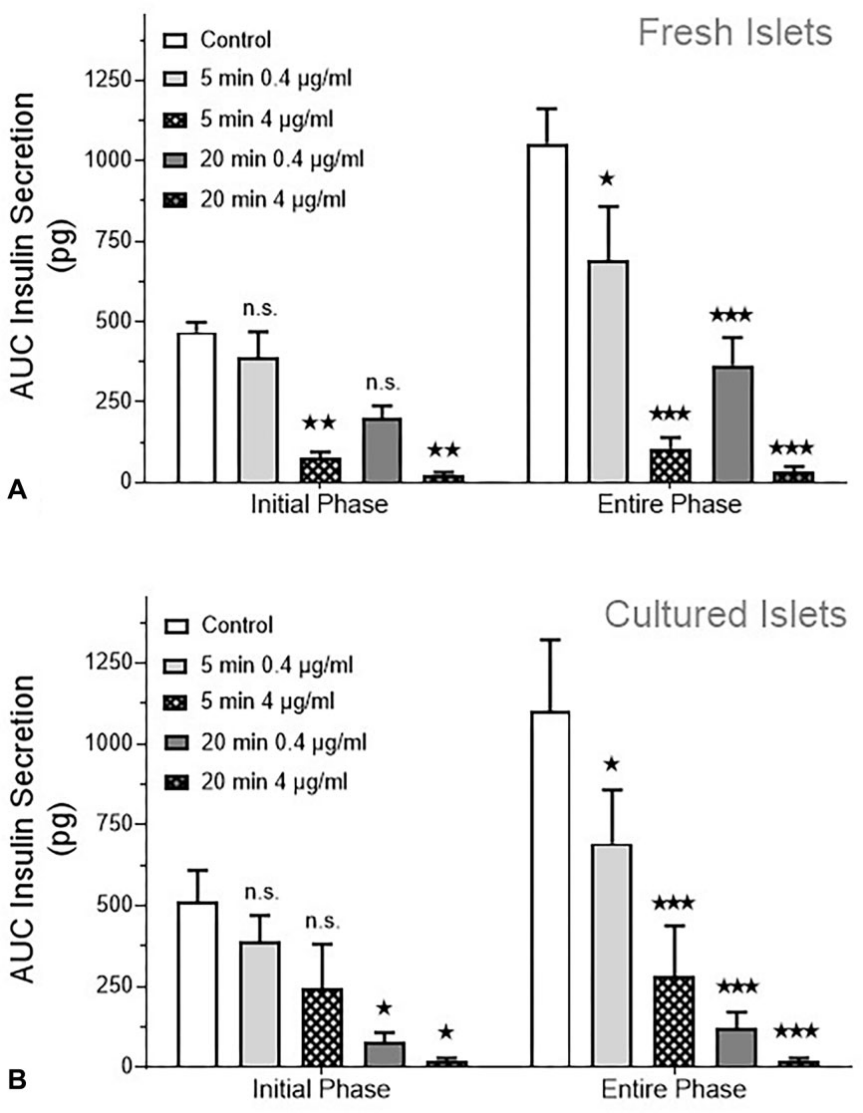
Statistical evaluation of the inhibitory action of oligomycin. The inhibitory effect of oligomycin on fresh islets (A) and cultured islets (B) as depicted in Figure 2 was quantified by calculating the integral of insulin released by K^+^ depolarization above the prestimulatory rate (“entire phase”). The release during the first 10 min of stimulated secretion (from 80 to 90 min) is given as “initial phase.” The significant difference from control was calculated by 2-way ANOVA with Bonferroni’s correction, 1, 2, or 3 asterisks stand for *P* < .05, *P* < .01, *P* < .001, respectively, n.s. for non-significant. Both the duration of oligomycin pre-exposure and the phase of stimulated secretion contributed significantly to the degree of inhibition, but the interaction between these parameters was only significant for fresh islets.

For the quantitative evaluation, the integral of secretion (AUC) during the exposure to 40 mm KCl was calculated (Figure 3). The 2-way ANOVA indicated that both, the intensity of exposure to oligomycin (duration of pre-exposure together with the oligomycin concentration) and the kinetics of depolarization-induced secretion (entire exposure to 40 mm KCl or initial phase only) contributed to the variation. However, the contribution of phase was less relevant for the cultured islets, and an interaction between the parameters existed only in fresh islets. So, both concentrations of oligomycin achieve a high degree of inhibition, but the initial phase was more strongly affected by the higher concentration.

**Figure 4.**
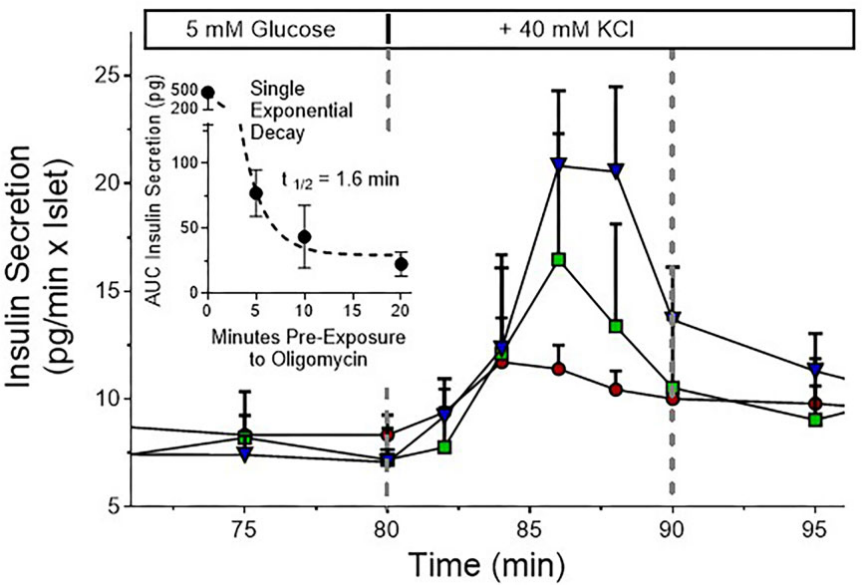
Decrease of the initial secretory response to K^+^ depolarization by prolonging the oligomycin pre-exposure period. The figure is a detail of Figure 2B with expanded scales of time and insulin secretion. The inset shows the integrals (AUC) of insulin secretion of these 3 secretory responses and of the pertaining control from 80 to 90 min as function of the oligomycin pre-exposure period. The decrease was significant (*P* < .001, Brown-Forsythe one-way ANOVA) and could be fitted with a mono-exponential decay function with a half-time of 1.6 min.

Based on these data, the time-dependent effect of 4 µg/mL oligomycin on the initial phase in fresh islets was analyzed in more detail (Figure 4). In addition to the 3 periods of oligomycin pre-exposure (5, 10, and 20 min), the control was included as time point zero. The highly significant decrease (one-way ANOVA) could be fitted with a single exponential function with a half-time of 1.6 min. In other words, the amount of insulin releasable by 40 mm KCl diminishes with this kinetic when a nearly instantaneous block of ATP generation is assumed.

### Reduction of the Adenine Nucleotide Content by Oligomycin

**Figure 5.**
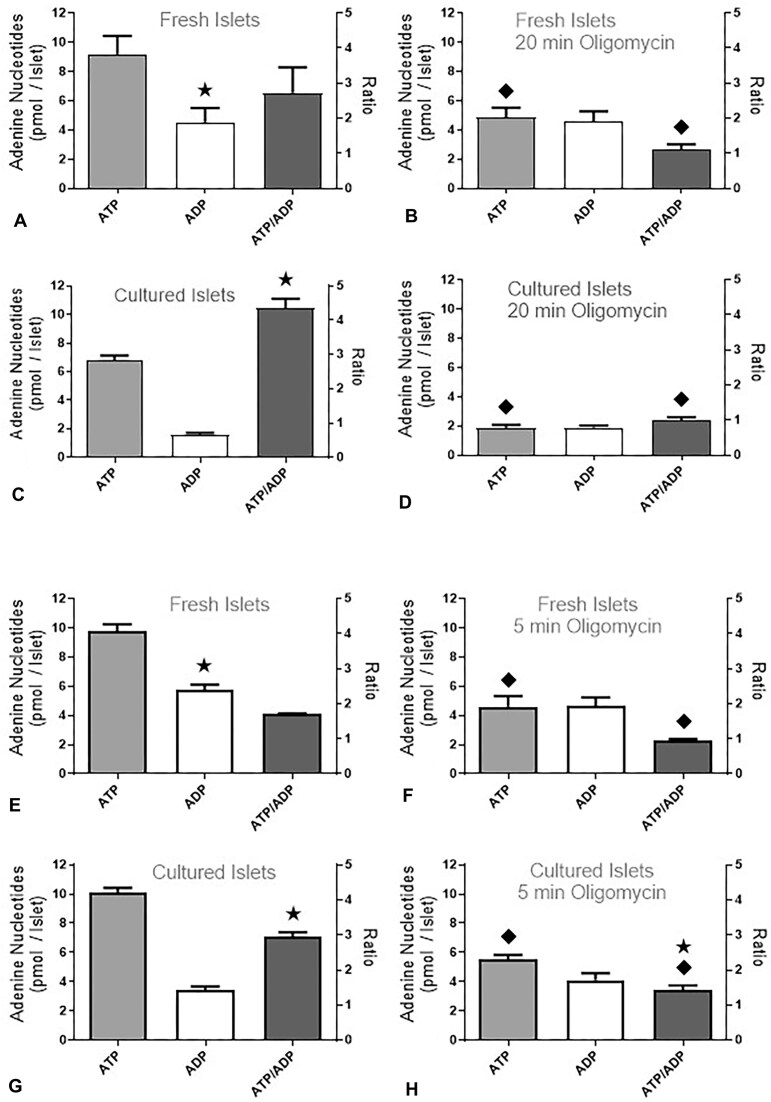
Effect of oligomycin on the adenine nucleotide content of statically incubated islets. Fresh or cultured islets were incubated for 60 min (Figure 5A and C) or 75 min (Figure 5E and G) in KR-medium with 5 mm glucose; thereafter, the contents of ATP and ADP were measured. Alternatively, 4 µg/mL oligomycin was added at 60 min, and the incubation was continued for another 20 min (Figure 5B and D) or added at 75 min with subsequent incubation for 5 min (Figure 5F and H). The asterisks denote significant differences between fresh and cultured islets (*P* ˂ .05), the rhombus symbols denote significant (*P* ˂ .05) differences between control islets (A, C, E, and G) and the corresponding oligomycin-exposed islets (B, D, F, and H). The values are means ± SEM of 6–8 experiments.

The ATP and ADP contents of fresh and cultured islets were measured after static incubation (Figure 5). The incubation protocol was designed to mimic the perifusion sequence prior to the K^+^ depolarization. After 60 min incubation at 5 mm glucose, a significantly higher ATP/ADP ratio existed in cultured islets than in fresh islets. This was mainly due to a higher content of ADP in the fresh islets (Figure 5A and B). The subsequent 20 min exposure to 4 µg/mL oligomycin caused a significant decrease of ATP but no increase of ADP in either islet preparation (Figure 5C and D). As the consequence, the ATP/ADP ratios of fresh and cultured islets were close to one and no longer significantly different. After 75 min incubation at 5 mm glucose, a significantly higher ATP/ADP ratio existed in cultured islets than in fresh islets. Again, this was mainly due to a higher content of ADP in the fresh islets (Figure 5E and F). The subsequent 5 min exposure to 4 µg/mL oligomycin caused a similarly strong decrease of ATP as the 20 min exposure (again no increase of ADP), this time, however, a significantly higher ATP/ADP ratio in cultured than in fresh islets persisted (Figure 5G and H). So, in freshly isolated islets a maximal inhibitory effect is achieved within 5 min by 4 µg/mL oligomycin.

### No Inhibition of the Depolarization-induced Increase of the Cytosolic Ca^2+^ Concentration ([Ca^2+^]_i_) Increase by Oligomycin

**Figure 6.**
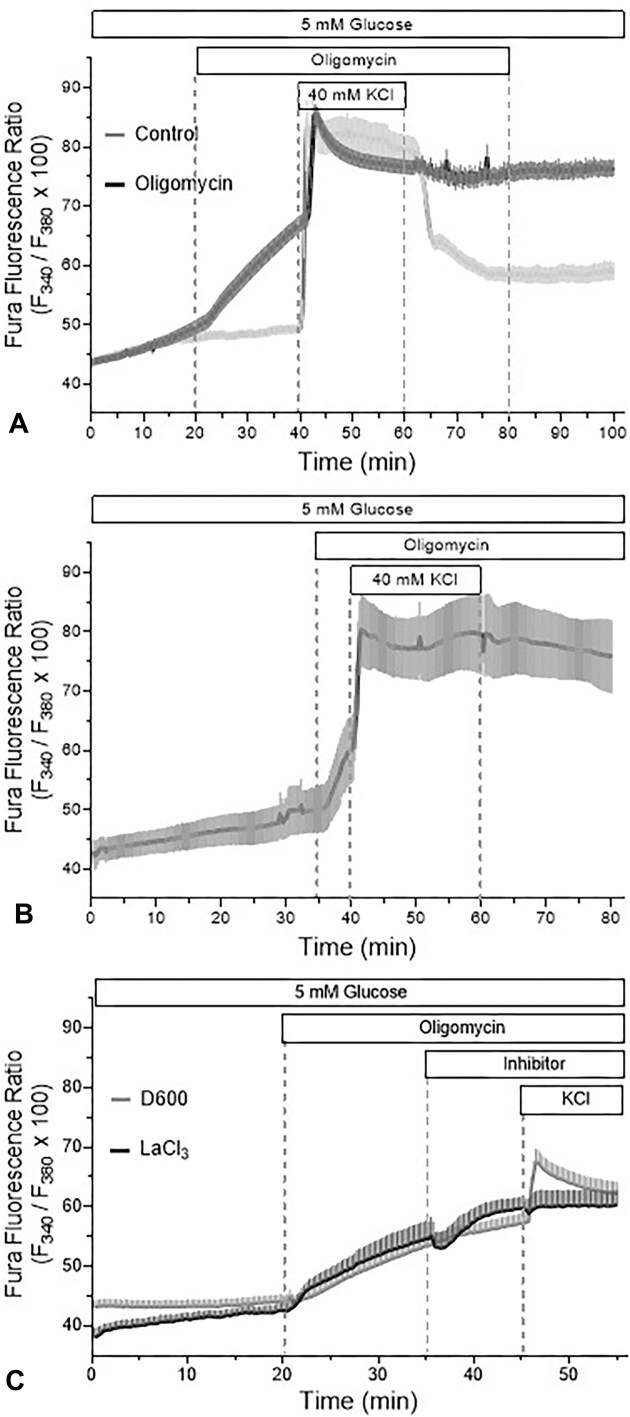
No effect of oligomycin on the depolarization-induced Ca^2+^ increase. Cultured islets were loaded with Fura-2 LeakRes (AM) and perifused with KR-medium (5 mm glucose). Before raising the K^+^ concentration from 5.9 to 40 mm, 4 µg/mL oligomycin was present for 20 min (A) or 5 min (B) and for 20 min after lowering back to 5.9 mm. Note the irreversibility of the Fura ratio increase during the washout of oligomycin (A). The mechanism underlying the Ca^2+^ increase was assessed by the addition of either 50 µM D 600 or 2 mm LaCl_3_ (C). The values are means ± SEM of 5–7 experiments.

To clarify whether the inhibition of the depolarization-induced insulin secretion by oligomycin involves alterations of the Ca^2+^ signal, the [Ca^2+^]_i_ of perifused islets was measured (Figure 6). 4 µg/mL oligomycin steadily increased the [Ca^2+^]_i_ after a lag time of about 2 min. The depolarization by 40 mm KCl after 20 min of oligomycin pre-exposure increased [Ca^2+^]_i_ further and established the same maximal value as the depolarization 40 mm KCl under control condition (Figure 6A). Remarkably, the depolarization-induced increase of [Ca^2+^]_i_ was not only irreversible in the presence of oligomycin, but also when it was washed out. When oligomycin was present for only 5 min prior to the K^+^ depolarization, the level of [Ca^2+^]_i_ prior to the K^+^ depolarization was lower than after 20 min of exposure but in principle the same reaction pattern was observed (Figure 6B). The increase of [Ca^2+^]_i_ by oligomycin could not be reversed by inhibitors of Ca^2+^ influx, D600 or LaCl_3_ (Figure 6C). In contrast, the [Ca^2+^]_i_ increase by 40 mm KCl was strongly reduced by 50 µM D600 and was suppressed by 2 mm LaCl_3_ (Figure 6C). In conclusion, the [Ca^2+^]_i_ increase by oligomycin is neither dependent on Ca^2+^ influx nor does it interfere with the generation of the Ca^2+^ signal for exocytosis.

### Effect of Oligomycin on the Plasma Membrane Potential and Transmembrane Currents

**Figure 7.**
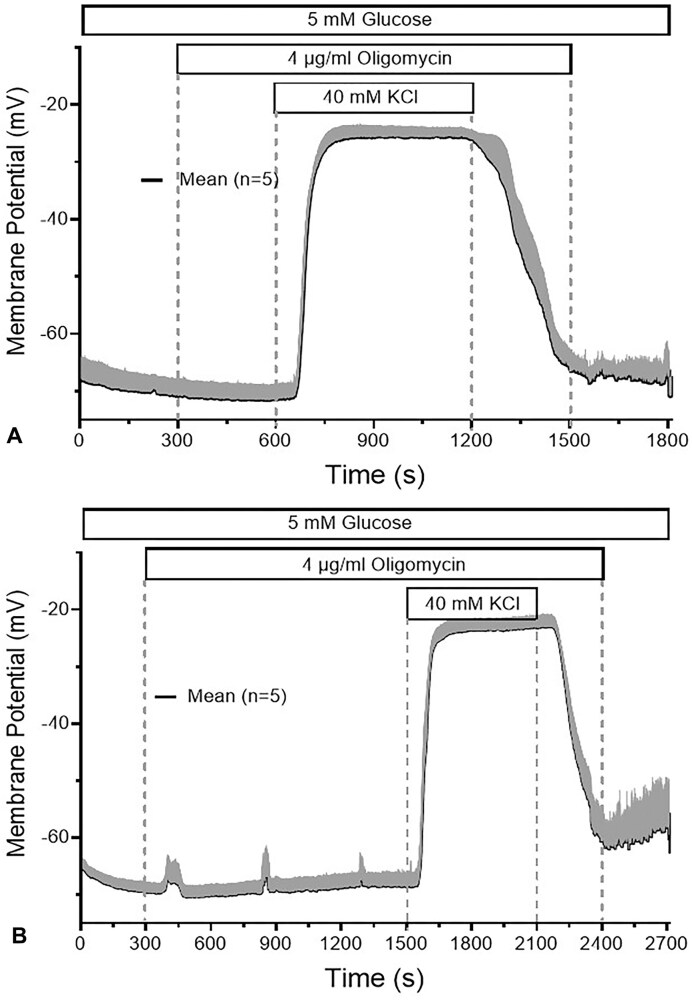
No effect of oligomycin on the resting membrane potential and the depolarization amplitude by 40 mm KCl. The membrane potential of 1d-cultured primary beta cells was measured using current clamp in the perforated-patch configuration. Before raising the K^+^ concentration in the extracellular solution from 5.9 to 40 mm, 4 µg/mL oligomycin was present for 5 min (A) or 20 min (B). Note that neither the resting membrane potential nor the depolarization amplitude was affected by oligomycin. The values are means ± SEM of 5 experiments.

**Figure 8.**
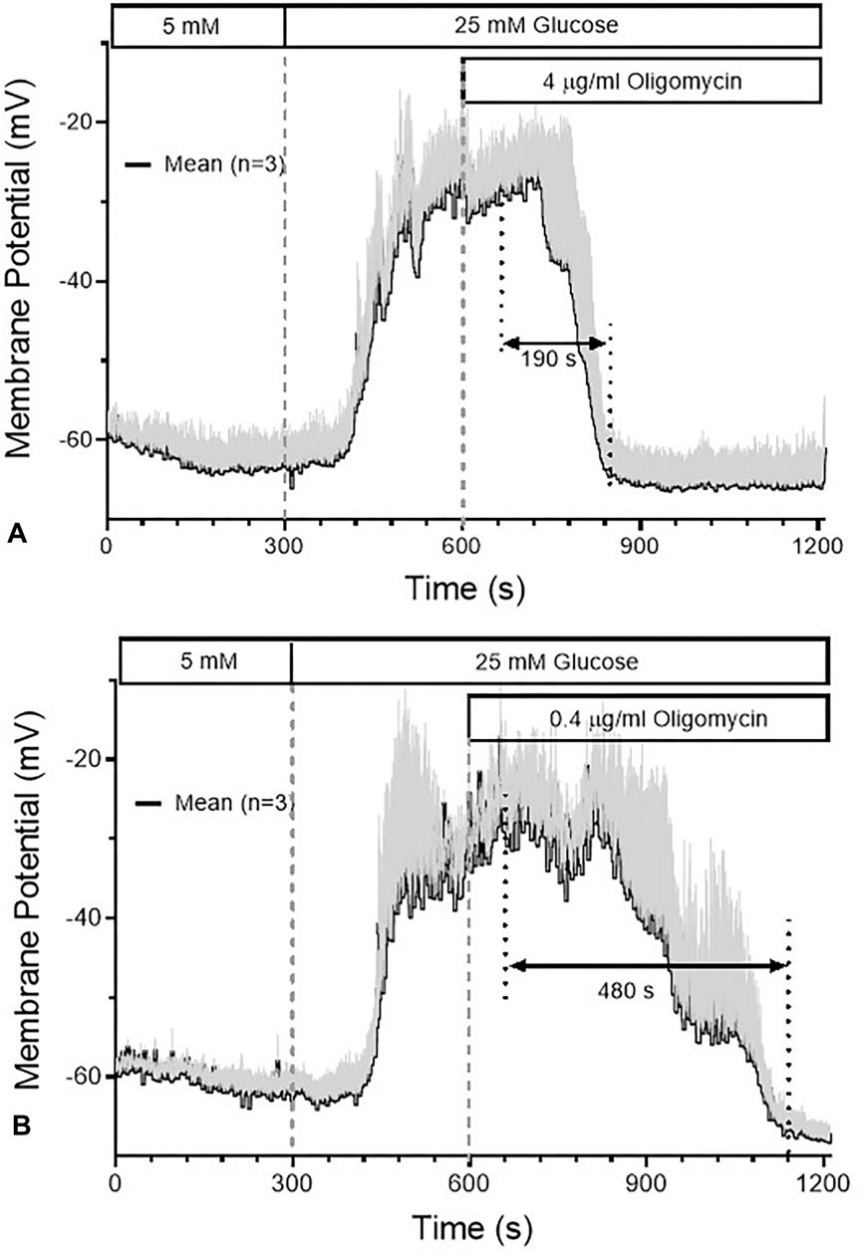
Concentration-dependent reversal of the glucose-induced depolarization by oligomycin. The membrane potential of 1d-cultured primary beta cells was measured using current clamp in the perforated-patch configuration. The depolarization was induced by raising the glucose concentration in the extracellular solution from 5 to 25 mm. After 5 min of perifusion with 25 mm glucose, oligomycin was added at 4 µg/mL (A) or 0.4 µg/mL (B). Note the slower time course of repolarization with the lower concentration. The values are means ± SEM of 3 experiments.

**Figure 9.**
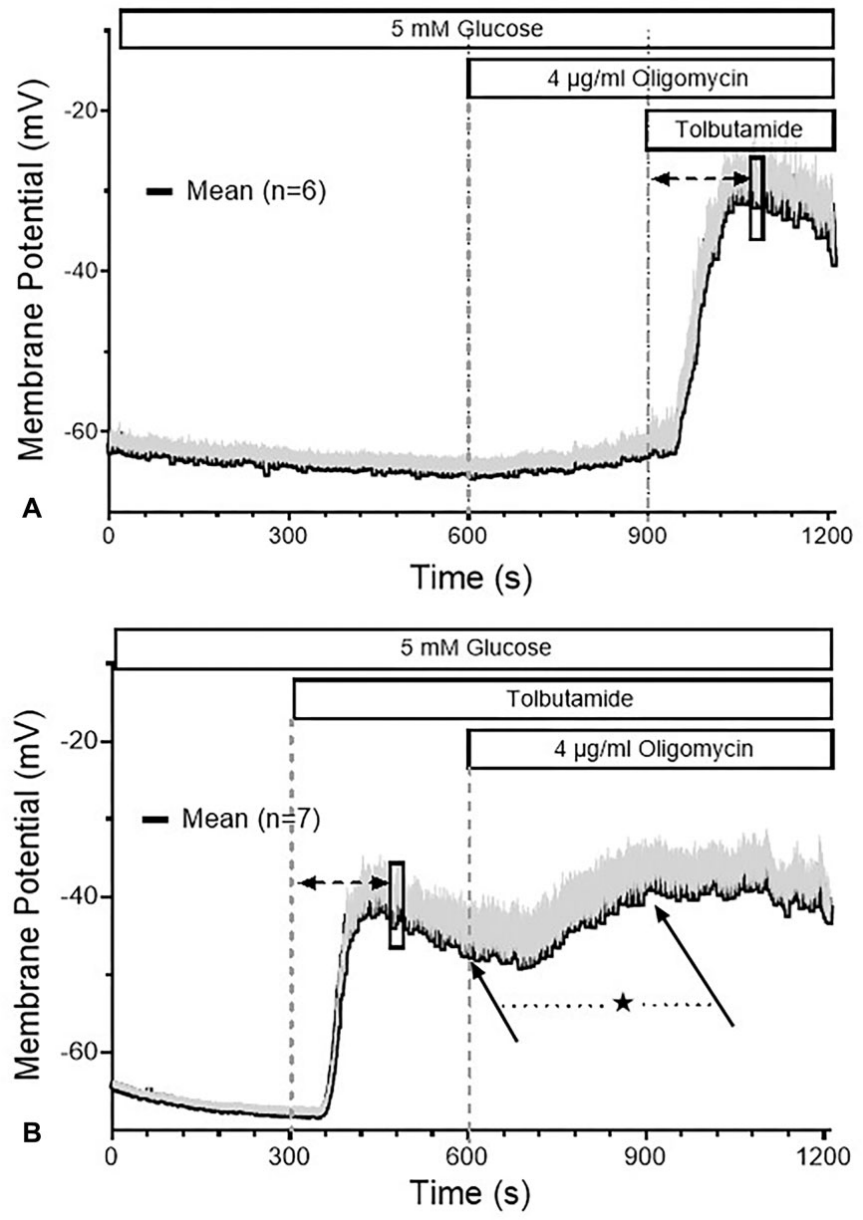
No inhibition by oligomycin of the depolarizing effect of the K_ATP_ channel blocker, tolbutamide. The membrane potential of 1d-cultured primary beta cells was measured using current clamp in the perforated-patch configuration. Before adding 500 µM tolbutamide to the extracellular solution, 4 µg/mL oligomycin was present for 5 min (A). Note the significant further depolarization when 4 µg/mL oligomycin was added 5 min after the addition of 500 µM tolbutamide (B, arrow at 600 s vs. arrow at 900 s, *P* = .02, paired 2-sided *t*-test). This conforms with the significantly stronger amplitude of depolarization by tolbutamide in (A) as compared with (B) after 3 min of exposure (indicated by the rectangles, *P* < .001, paired 2-sided *t*-test). The values are means ± SEM of 6–7 experiments.

At 4 µg/mL, oligomycin did not affect the resting membrane potential of single beta cells. The depolarization amplitude in response to 40 mm KCl was as theoretically expected (40 mV) and did not differ between pre-exposure times of 5 and 20 min (Figure 7A and B). In contrast, the depolarization caused by increasing the glucose concentration from 5 to 25 mm was completely reversed within 3 min (Figure 8A). Even at 0.4 µg/mL a complete repolarization was achieved; however, the velocity was about 3-fold slower than the reversal produced by the 10-fold higher concentration (Figure 8B). The depolarization by the pharmacological blocker of the K_ATP_ channels, 500 µM tolbutamide, was not antagonized by pretreatment with 4 µg/mL oligomycin (Figure 9A), rather, a moderate further increase of depolarization was noted when this concentration of oligomycin was added in the presence of tolbutamide (Figure 9B). K_ATP_ currents evoked by small hyper- and depolarization steps (10 mV) around the resting membrane potential (−70 mV) showed no increase, but a small, non-significant decrease during prolonged exposure (data not shown). These data suggest that oligomycin has no direct effect on K_ATP_ channels but acts via diminution of the ATP/ADP ratio. At 4 µg/mL the critically low value opening the channels is apparently reached after 1 min (Figure 7A).

## Discussion

It has been shown earlier that the stimulation of insulin secretion by nutrient secretagogues comprises the closure of the K_ATP_ channel and an additional step that requires activity of the respiratory chain.^19,20,25^ Since the inhibition of the oxidative phosphorylation abolished the secretion elicited by Ca^2+^ influx, this energy-requiring step was located distal to the influx of Ca^2+^.^19,20,32^ However, this conclusion is not compatible with the SNARE hypothesis of triggered exocytosis, which proposes that Ca^2+^ binding to synaptotagmin at the vesicle or granule membrane is the immediate signal for fusion^12^ (see however ^33^ ) The present observations suggest that a continuously running oxidative phosphorylation is necessary to keep the exocytotic machinery in a fusion-ready state. Thus, in the sequence of events, the energy-requiring step is located prior to the effect of Ca^2+^.

Beta cells contain a large number of insulin granules, the values range between 5000 and 10 000 granules per cell.^34,35^ During stimulation, the exocytotic events per cell range, depending on methodology, between 14 granules per second^36^ and 13 granules per minute.^37^ The amount of insulin released during the entire first phase of glucose-induced secretion represents less than 5% of the cellular insulin content.^38^ Thus, it is generally accepted that only a minority of the insulin granules within the beta cell is fusion-competent.^13–15^ Since the synthesis of insulin and the formation of granules depend on nutrient availability but not on Ca^2+^ influx from the extracellular space^39^, and since the conditions prevailing at the time of granule biogenesis affect the probability that newly synthesized insulin is preferentially released,^40^ it is implicitly assumed that exocytosis-determining characteristics are conferred upon the granules at the time of formation.

On the other hand, it is also generally accepted that, in addition to the spatial proximity to the plasma membrane an additional qualitative property has to be conferred on the granule to establish the ultimate state of fusion competence, which is usually described as the granule being “docked and primed.”^15^ Such granules are considered to make up the “readily releasable pool,” which is depleted during the first phase of glucose-stimulated secretion and is gradually replenished from the “reserve pool” during the second phase.^15^ While the number of reactions involved in the priming process continues to grow, it is still assumed that fusion competence, once reached, is a stable property of the granules awaiting the fusion signal.^41^

Therefore, it could be expected that the inhibition of oxidative phosphorylation abolishes energy-dependent processes like granule transport and priming but leaves the pool of fusion-competent granules unchanged. However, the prolonged absence of glucose prior to the stimulation had a profound effect on the depolarization-induced secretion, as is shown by the moderate transient secretion caused by 15 mm KCl, which could not be increased by raising KCl to 40 mm (see Figure 1). Rather, the secretion rate even receded, suggesting that the amount of releasable insulin was already exhausted.

A much slower diminution of releasable insulin was observed when secretion pulses were elicited by repeated pulses of 30 mm KCl in the absence of glucose, suggesting a much larger pool size.^42^ However, endogenous fuels of the beta cell permit a temporal ATP supply even in absence of glucose.^43^ Thus, a prolonged absence of exogenous nutrients is needed to reveal the amount of insulin that can be secreted with minimal contribution of the oxidative phosphorylation. Of note, such an absence prior to stimulation with 30 mm glucose transformed the secretion kinetics into a continuously ascending secretion without a first phase.^44^ While hyperosmolarity may contribute to the first phase, ^45^ its effect seems also to be energy-dependent.

The question why the absence of glucose has such a profound effect that even the secretory response to 40 mm KCl (which is a stimulus of supraphysiological strength ^26,27^) was lost, was addressed by systematically lowering the mitochondrial ATP production. Oligomycin was chosen as a pharmacological tool since it lowers the ATP production by binding to a specific target, the oligomycin-sensitivity-conferring peptide of the F_1_F_O_ ATPase, and thereby inhibiting the proton flow through the ATPase.^46^ To distinguish effects representing the pharmacokinetics of the compound from those representing the pharmacodynamics, oligomycin was used at 2 different concentrations on 2 different preparations, freshly isolated islets and islets cultured for 1 day.

It became clear that the oligomycin concentration that is typically used (4 µg/mL, equivalent to ca. 5 µM) is supramaximal in the sense that a complete inhibition of secretion and reversal of glucose-induced depolarization can also be achieved by a 10-fold lower concentration but requires more time. The supramaximal concentration also explains the marked asymmetry in the kinetics of action: a fast onset upon introduction and a very slow offset upon washout (see Figure 6). The same conclusion was reached in an earlier investigation on the mitochondrial ATP production in hepatocytes, which gave a value of as low as 0.01 µg/mL of oligomycin to achieve a complete suppression when the exposure time was prolonged to 2 h.^47^

It has previously been shown that fresh and cultured islets differ in their secretory response to nutrient stimuli but not to purely depolarizing stimuli.^48^ These observations were confirmed here by the closely similar KCl-induced secretion of the control perifusions. It is remarkable that the inhibition of ATP synthesis by 4 µg/mL oligomycin reduced the ATP/ADP ratio of fresh and cultured islets to the same extent after 20 min but was more efficient in fresh islets than in cultured islets when the exposure time was only 5 min. This difference may be due to the larger proton leak of the mitochondria in fresh islets,^49^ which diminishes the coupling efficiency of the oxidative phosphorylation. The different reduction of the ATP/ADP ratio fits to the stronger inhibition of secretion in fresh than in cultured islets under this condition.

The analysis of the amount of insulin released during the K^+^ depolarization showed that the effect of oligomycin is time-dependent in 2 respects: the degree of inhibition increases during the depolarization phase, and the degree of inhibition of the initial phase increases particularly strong with the duration of pre-exposure. The pronounced susceptibility of the fresh islets to the action of the higher oligomycin concentration is expressed by the surprisingly short half-time value of 1.6 min for the reduction of the initial response. This value is in the same range as the lag time before the [Ca^2+^]_i_ increase by oligomycin and the time required for the half maximal reversal of glucose-induced depolarization (see below). Thus, there is a fast time-dependent reduction of releasable insulin when the oxidative phosphorylation is inhibited. Further research will have to clarify whether the decreasing levels of ATP are associated with a different pattern of granule localization and mobility.^50^

As a consequence of its mechanism of action, oligomycin does not decrease the mitochondrial membrane potential, in contrast to many other inhibitors of the oxidative phosphorylation like azide, uncouplers, or the inhibitors of the respiratory complexes I and III.^49^ Thus, unlike these inhibitors, oligomycin does not induce a Ca^2+^ efflux from the mitochondria (Gaus and Rustenbeck, unpublished), which would be a possible mechanism for the slow increase of [Ca^2+^]_i_. Since the increase was neither affected by D600 nor by lanthanum (see Figure 5C), influx of Ca^2+^ from the extracellular space is also unlikely. Lanthanum, which in contrast to D600 completely suppressed the [Ca^2+^]_i_ increase by 40 mm KCl, was used since it does not enter beta cells as has been shown earlier by the ^45^Ca^2+^ technique^51,52^ and, in contrast to other ionic Ca^2+^ channel blockers, does not quench the Fura fluorescence signal.^53^

So, it can be concluded that the oligomycin-induced [Ca^2+^]_i_ increase likely results from the imbalance between intracellular non-mitochondrial Ca^2+^ release and ATP-dependent sequestration and extrusion. Such ATP-dependent Ca^2+^ transport systems are the SERCA pump of the endoplasmic reticulum and the PMCA pump of the plasma membrane,^54,55^ but also the Na-K^+^ ATPase, which, when arrested, indirectly raises [Ca^2+^]_i_.^56^ The continued inhibition of these transport systems by lack of ATP explains that the depolarization-induced [Ca^2+^]_i_ increase was not reversible, even when oligomycin was no longer present in the perifusion medium.

The observation that 4 µg/mL oligomycin affected neither the resting membrane potential nor the extent of depolarization by 40 mm KCl conforms to the above conclusion that the [Ca^2+^]_i_ increase by 40 mm KCl was not altered by the presence of oligomycin. The complete reversal by oligomycin of the depolarization induced by 25 mm glucose is a logical consequence of the opening of the K_ATP_ channels by decreasing ATP levels. Thus, a critically low ATP/ADP ratio in the submembrane space is reached within one or 2 min. The lack of repolarization by oligomycin in the presence of tolbutamide (there was even a slight but significant further depolarization in accordance with the observation by Zunkler et al. ^31^) confirms that the opening of the K_ATP_ channels in the presence of high glucose originates from the inhibition of oxidative phosphorylation, whatever the precise mechanism of signal transduction may be.^57,58^ The observation that the 10-fold lower concentration of oligomycin is also able to achieve a complete repolarization, albeit with slower kinetics of action, is also relevant. By analogy, it can be assumed that the weak inhibition of the initial secretion phase by this concentration is due to the slower build-up of the critical concentration and does not indicate a lesser energy dependence of the initial phase.

The reason why ATP is not only needed to establish fusion competence but has to be continuously supplied may lie in the activity of the vacuolar-type H^+^-ATPase (V-ATPase) of the insulin granules. This ATPase, although similar to the F_1_F_O_ ATPase of the mitochondria, is not inhibited by oligomycin.^59^ It is responsible for the acidification of the granule interior, required for the maturation of the granule content,^60^ and was suggested to be involved in the ATP-dependent priming of the granules.^61,62^ Moreover, the V-ATPase has been implicated in the membrane fusion in multiple cellular systems, but it is currently unclear whether this is a consequence of the acidification or an additional function.^63,64^ In view of the large number of granules, it is tempting to speculate that the V-ATPase contributes to the ATP sink, which decreased the ATP/ADP ratio in beta cells but not in alpha cells upon lowering of the ambient glucose concentration.^65^

In summary, the present study shows that the oxidative phosphorylation has a direct effect on the amount of releasable insulin, which quickly decreases when the ATP production is blocked. Thus, the fusion competence of the insulin granules or of the entire exocytotic machinery appears to be a reversible state. A limitation of the study is that the inhibition of oxidative phosphorylation reduces the ATP/ADP ratio to unphysiological low levels. The same reasoning applies to the prolonged absence of glucose. Therefore, the energetic requirement to keep the granules in a fusion-competent state may only play a permissive role and may not be involved in the regulation of stimulated secretion, such as exerted by the incompletely understood signals of metabolic amplification.^22^ However, it may be relevant to keep the basal insulin secretion low during periods of low nutrient supply.

## Data Availability

All data relevant to evaluate the conclusions in the paper are present in the paper.
